# Chromosomal Instability as a Driver of Therapeutic Resistance and Phenotypic Plasticity in Non‐Small Cell Lung Cancer

**DOI:** 10.1111/jcmm.70867

**Published:** 2025-09-28

**Authors:** Dongjing Ma, Jinbin Wang, Keqin Gao

**Affiliations:** ^1^ School of Public Health Gansu University of Chinese Medicine Lanzhou Gansu China; ^2^ The Collaborative Innovation Center for Prevention and Control by Chinese Medicine on Diseases Related Northwestern Environment and Nutrition Gansu University of Chinese Medicine Lanzhou Gansu China

**Keywords:** chromosomal instability, non‐small cell lung cancer, phenotypic plasticity, therapeutic resistance

Dear Editor,

Chromosomal instability (CIN), defined as the continuous missegregation of chromosomes during mitosis, is a hallmark of many solid tumours, including non‐small cell lung cancer (NSCLC) [1]. CIN has traditionally been regarded as a consequence of oncogenic stress; however, recent findings indicate that CIN may actively contribute to tumour heterogeneity, immune modulation and resistance to therapy. This letter outlines emerging insights into the mechanistic involvement of CIN in NSCLC progression and suggests that its effects extend beyond passive genomic instability.

CIN has been linked to widespread aneuploidy and micronuclei formation, which frequently result from defective mitotic regulators such as BUB1, MAD2, Aurora B and STAG2 [2]. These abnormalities not only induce structural chromosomal rearrangements but also affect gene dosage and epigenetic landscapes. CIN‐induced genomic plasticity has been shown to facilitate the emergence of drug‐tolerant clones under therapeutic pressure, allowing rapid adaptation without the need for driver mutations [2]. Such adaptability may underlie the persistent resistance observed in targeted therapies for non‐small cell lung cancer, particularly those directed against EGFR and KRAS alterations [3].

In addition to its role in genomic rearrangement, CIN appears to influence transcriptional reprogramming. Recent studies suggest that prolonged mitotic stress can promote transitions to hybrid epithelial–mesenchymal phenotypes via nongenetic mechanisms [4]. This may involve transient chromatin accessibility during mitotic exit, enabling the expression of plasticity‐associated factors such as ZEB1 and SNAI2 [5]. These changes are associated with reduced sensitivity to kinase inhibitors and may contribute to phenotypic switching, a phenomenon increasingly recognised in treatment‐refractory NSCLC [6].

The immunological consequences of CIN also warrant consideration. Chromosome missegregation often results in the formation of cytosolic DNA fragments, which can activate the cyclic GMP–AMP synthase (cGAS)–stimulator of interferon genes (STING) pathway [7]. While this pathway initially elicits antitumour immunity, sustained activation has been associated with STING desensitisation, impaired interferon signalling, and the recruitment of immunosuppressive cells such as regulatory T cells [8]. These findings suggest that CIN may shape the tumour immune microenvironment in ways that impair immune checkpoint blockade efficacy in certain NSCLC subtypes [9].

Furthermore, CIN has been proposed as a therapeutic vulnerability. CIN‐high tumours are often dependent on intact DNA damage response (DDR) pathways, including the CHK1 and ATR signalling pathways, to mitigate replication stress. Inhibition of these pathways has been shown to induce mitotic catastrophe in preclinical models [10]. Similarly, therapies that target centrosome clustering or spindle checkpoint adaptation may exploit CIN‐associated fragility. Nonetheless, the clinical integration of CIN‐related biomarkers remains limited, and standardised quantification methods are lacking.

Although CIN is a widely observed feature in NSCLC, its functional roles in tumour adaptability, immune evasion and therapy resistance remain incompletely understood [11]. Clarifying how CIN intersects with transcriptional plasticity and immunological remodelling may provide new avenues for overcoming resistance. Therapeutic strategies that exploit CIN‐induced vulnerabilities, particularly in combination with immunotherapy or DDR inhibitors, represent promising directions for future research.

## Author Contributions

Jinbin Wang wrote the original draft. The conceptual framework of the letter was developed by Dongjing Ma and Jinbin Wang. Dongjing Ma and Keqin Gao contributed to the revision and critical editing of the manuscript.

## Conflicts of Interest

The authors declare no conflicts of interest.

## Data Availability

The data that support the findings of this study are available from the corresponding author upon reasonable request.

## References

[1] R. Hosea , S. Hillary , S. Naqvi , S. Wu , and V. Kasim , “The Two Sides of Chromosomal Instability: Drivers and Brakes in Cancer,” Signal Transduction and Targeted Therapy 9, no. 1 (2024): 75, 10.1038/s41392-024-01767-7. 38553459 PMC10980778

[2] D. T. Loughlin , “Every Patient,” Trends in Cancer 8, no. 4 (2022): 259–261, 10.1016/j.trecan.2022.02.001. 35249852

[3] S. Hobor , M. Al Bakir , C. T. Hiley , et al., “Mixed Responses to Targeted Therapy Driven by Chromosomal Instability Through p53 Dysfunction and Genome Doubling,” Nature Communications 15, no. 1 (2024): 4871, 10.1038/s41467-024-47606-9. PMC11176322 38871738

[4] K. J. Song , S. Choi , K. Kim , et al., “Proteogenomic Analysis Reveals Non‐Small Cell Lung Cancer Subtypes Predicting Chromosome Instability, and Tumor Microenvironment,” Nature Communications 15, no. 1 (2024): 10164, 10.1038/s41467-024-54434-4. PMC11585665 39580524

[5] S. E. Parfenyev , A. A. Daks , O. Y. Shuvalov , et al., “Dualistic Role of ZEB1 and ZEB2 in Tumor Progression,” Biology Direct 20, no. 1 (2025): 32, 10.1186/s13062-025-00604-3. 40114235 PMC11927373

[6] E. B. Gunnarsson , S. De , K. Leder , and J. Foo , “Understanding the Role of Phenotypic Switching in Cancer Drug Resistance,” Journal of Theoretical Biology 490 (2020): 110162, 10.1016/j.jtbi.2020.110162. 31953135 PMC7785289

[7] K. Yonesaka , T. Kurosaki , J. Tanizaki , et al., “Chromosomal Instability Is Associated With cGAS–STING Activation in EGFR‐TKI Refractory Non‐Small‐Cell Lung Cancer,” Cells 14, no. 6 (2025): 447. 40136696 10.3390/cells14060447 PMC11941500

[8] J. Li , M. J. Hubisz , E. M. Earlie , et al., “Non‐Cell‐Autonomous Cancer Progression From Chromosomal Instability,” Nature 620, no. 7976 (2023): 1080–1088, 10.1038/s41586-023-06464-z. 37612508 PMC10468402

[9] J. V. Alessi , X. Wang , A. Elkrief , et al., “Impact of Aneuploidy and Chromosome 9p Loss on Tumor Immune Microenvironment and Immune Checkpoint Inhibitor Efficacy in NSCLC,” Journal of Thoracic Oncology 18, no. 11 (2023): 1524–1537, 10.1016/j.jtho.2023.05.019. 37247843 PMC10913104

[10] X. Mao , N. K. Lee , S. E. Saad , and I. L. Fong , “Clinical Translation for Targeting DNA Damage Repair in Non‐Small Cell Lung Cancer: A Review,” Translational Lung Cancer Research 13, no. 2 (2024): 375–397. 38496700 10.21037/tlcr-23-742 PMC10938103

[11] Q. Li , W. Qian , Y. Zhang , L. Hu , S. Chen , and Y. Xia , “A New Wave of Innovations Within the DNA Damage Response,” Signal Transduction and Targeted Therapy 8, no. 1 (2023): 338, 10.1038/s41392-023-01548-8. 37679326 PMC10485079

